# M-plane GaN terahertz quantum cascade laser structure design and doping effect for resonant-phonon and phonon-scattering-injection schemes

**DOI:** 10.1038/s41598-023-38627-3

**Published:** 2023-07-14

**Authors:** Fan Ye, Yiyang Wang, Li Wang, Tsung-Tse Lin, Fantai Zeng, Yue Ji, Jinchuan Zhang, Fengqi Liu, Hideki Hirayama, Ke Wang, Yi Shi, Youdou Zheng, Rong Zhang

**Affiliations:** 1 School of Electronics Science and Engineering, Nanjing University, 163 Xianlin Street, Qixia District, Nanjing, 210046 China; 2 RIKEN Center for Advanced Photonics, THz Quantum Device Team, 519-1399 Aramaki-aza Aoba, Aoba-ku, Sendai, 980-0845 Japan; 3 Key Laboratory of Semiconductor Materials Science, Institute of Semiconductors, Chinese Academy of Sciences, 35 Tsinghua East Road, Haidian District, Beijing, 100083 China

**Keywords:** Electrical and electronic engineering, Quantum cascade lasers

## Abstract

Non-polar m-plane GaN terahertz quantum cascade laser (THz-QCL) structures have been studied. One is traditional three-well resonant-phonon (RP) design scheme. The other is two-well phonon scattering injection (PSI) design scheme. The peak gains of 41.8 and 44.2 cm^−1^ have been obtained at 8.2 and 7.7 THz respectively at 300 K according to the self-consistent non-equilibrium Green’s function calculation. Different from the usual GaAs two-well design, the upper and lower lasing levels are both ground states in the GaN quantum wells for the PSI scheme, mitigating the severe broadening effect for the excited states in GaN. To guide the fabrication of such devices, the doping effect on the peak gain has been analyzed. The two designs have demonstrated distinct doping density dependence and it is mainly attributed to the very different doping dependent broadening behaviors. The results reveal the possibility of GaN based THz-QCL lasing at room temperature.

## Introduction

Terahertz (THz) quantum cascade lasers (QCLs) have attracted wide attention for extensive applications in the fields of spectroscopy, chemical detection, imaging, and astronomy^1–4^. The operating temperature remains a central issue which confines THz-QCLs in the laboratory environment due to the necessity of cryogenic cooling. Recently a two-well THz-QCL based on resonant-phonon (RP) scheme operating at 250 K has been achieved^5^, which can be integrated into a compact and portable THz-QCL system with a thermoelectric cooler. However, room temperature operation is still challenging. On the other hand, the lasing frequency of the traditional GaAs based THz-QCLs has been limited between 0.8 and 5.4 THz^6^. GaAs/AlGaAs THz-QCLs are severely restrained in the frequency gap (5.5–12 THz) for solid-state coherent light sources, mainly because the forbidden Reststrahlen bands of the GaAs materials are approximately 30–50 meV and the non-radiative transitions via longitudinal optical (LO) phonon are much faster than the radiative transition^7^. In order to circumvent these limitations, GaN materials are considered as prospective candidates due to its much larger LO-phonon energy (~ 90 meV) with respect to GaAs (~ 36 meV). Such a property can contribute to the suppression of thermal back-filling and depopulation of the upper lasing level (ULL) through thermally activated LO-phonon transitions^8–12^. Theoretically, GaN based THz-QCLs would simultaneously fulfill the gap frequency and elevate operating temperature.

Due to the large lattice and thermal mismatch between a nitride and its substrate, growth technologies of the GaN/AlGaN system are not as mature as that of GaAs and InP systems. Since the polarization field is extremely strong, 0.1–1 MV cm^−1^, the conduction band profile of a polar (c-plane) GaN/AlGaN superlattice is sawtooth like^9^. The top of its quantum barrier and the bottom of its quantum well are triangles, which makes the design and simulation much more complicated than traditional compound semiconductor material systems. Since the alignment of the subband states is crucial for both injection and extraction processes, precise determination of the subband state energies is a must. However, the polarization charges at interfaces of GaN/AlGaN hetero-structures introduce a large uncertainty to the energy band profile. It is attributed to the difficulties in precise quantification of polarization charge densities. They are determined by spontaneous and piezoelectric polarization, which are directly related to the AlN molar fraction of the AlGaN barriers and residual strain in the active layers^13^. Moreover, the errors of spontaneous polarization and piezoelectric coefficients of III-nitride systems are still large, not to mention the interface roughness induced distortion of the polarization charge distribution profile, as Ref.^9^ discussed. Although our previous works reported narrow THz emission and designed some improved structures, it is still very challenging to fabricate a working GaN THz QCL based on polar GaN technology^11^.

An alternative choice is the non-polar plane GaN/AlGaN multiple quantum well structures as active layers^14^, in which the hetero interface polarization charge is zero. Without polarization, the energy band profile of a GaN QCL would be similar to a traditional GaAs QCL. In this way, the uncertainties induced by the polarization are completely avoided, leading to similar design considerations and much higher tolerance to the barrier composition deviation during epitaxial growth. In this report, we adopt m-plane GaN/AlGaN MQWs to design GaN THz QCLs. The strong electron-LO-phonon (e-phonon) coupling strength of GaN, with a Fröhlich constant 16 times as that of GaAs, would induce serious broadening for the related subbands^9^, and thus one must carefully consider this effect. Preferable designs with comprehensive calculations are urgently needed in order to guide future epitaxial growth and fabrication of a practical GaN THz QCL device.

We have theoretically studied M-plane GaN/AlGaN THz-QCLs with two design schemes, namely, the three-well RP scheme and the two-well phonon scattering injection (PSI) scheme. Nonpolar GaN (m-plane) quantum wells are chosen to alleviate the effect of polarization charge at the hetero interfaces. Non-equilibrium Green’s functions (NEGF) method has been utilized to analyze the carrier distribution, current density and gain properties of GaN/AlGaN THz-QCLs. NEGF calculations are processed through the Nextnano.negf tool by self-consistently solving Poisson and Schrödinger equations^15–19^. This method takes broadening, elastic scattering (ionized impurities, interface roughness, and alloy disorder etc.) and inelastic scattering (optical and acoustic phonons) all into account^9–11, 15–22^. Doping is an important parameter and has been demonstrated to strongly influence the carrier transport, gain, emission frequency, and waveguide loss as well. In some extreme cases, high doping density would even change the shape the energy band profiles and thus breaks the subband alignment, which is crucial for lasing operation^23–29^.

With the assistance of NEGF method, two possible GaN based THz-QCLs working at room temperature are investigated. We only consider the ground state of each well and the first excited state of the widest well, including related lasing, extraction and phonon scattering levels. The three-well RP and two-well PSI structures emitting at 8.2 and 7.7 THz at 300 K demonstrate maximum peak gain of 40.1 and 44.2 cm^−1^, which are above the cavity loss (20–30 cm^−1^) and feasible for environment without a cooler. The effect of doping has been studied across a wide range of sheet doping density, 2–16 × 10^10^ cm^−2^.

## Results and discussions

### Three-well RP scheme

**Figure 1:**
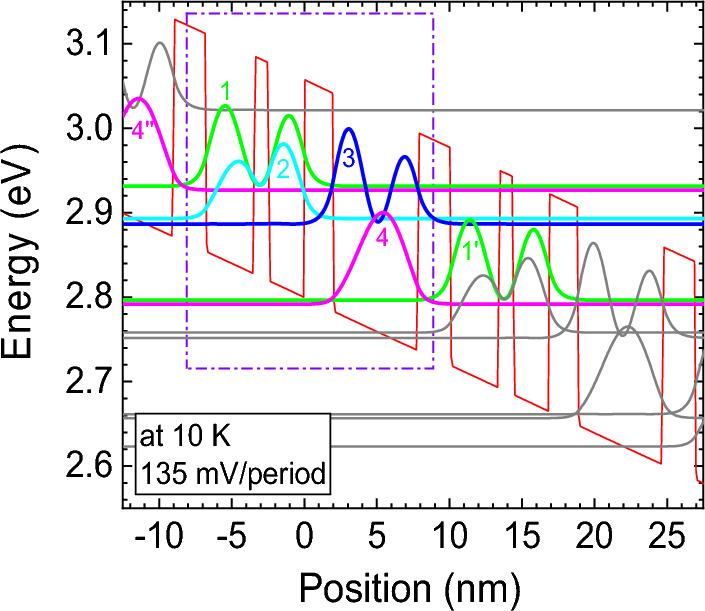
Conduction band diagram and envelope wavefunctions of TB subbands for the three-well RP GaN THz-QCL design. The operating bias is 135 mV/period at 10 K. A tight-binding mode is applied at injection barrier between two adjacent periods. The thicknesses of the quantum well (GaN) and barrier (Al_0.15_Ga_0.85_N) for each period are 22.4/33.3/10/24.5/20/58.4 Å (barrier thicknesses in bold). The periodic sheet doping density is set to 6 × 10^10^ cm^−2^ with the 5-nm-wide doping in the middle of the widest well.

We first consider the three-well RP THz-QCL design^30–33^, in which Al_0.15_Ga_0.85_N and GaN are employed as the barrier and well, respectively. The layer sequence in one period from the injection barrier is 22.4/33.8/10/25/20/58.4 Å, where the barriers are in bold fonts and the Si doped well is underlined. The sheet doping density is 6 × 10^10^ cm^−2^ and the doping region is 5 nm wide in the middle of the widest well to relieve the effect of interface roughness. Assuming the conduction/valence band offset (CBO/VBO) ratio of 3:1^34^, a CBO of 258 meV is obtained, including strain effect. Other material parameters are from Ref.^35^. The conduction band profile and wavefunctions of Tight-Binding (TB) subbands are shown in Fig. 1 at the designed bias of 135 mV/period.

The carriers are injected into the ULL 1 from the injector level (IL) 4’″ through resonant tunneling, with an anticrossing energy of 2.4 meV. There is a trade-off when choosing the proper thickness of the injector barrier, since it not only should be thin enough to strengthen resonant tunneling but also cannot be too thin for the purpose of preventing the non-selective injection from IL 4″ to lower lasing level (LLL) 2. Radiative transition takes place between ULL 1 and LLL 2 with an oscillator strength (${f}_{21}$) of 0.80 given by ${f}_{21}=4\uppi {m}_{e}^{*}{v}_{21}{z}_{21}^{2}/\mathrm{\hslash }$, where ${m}_{e}^{*}$ is the electron effective mass and equals to 0.20 ${m}_{e}$^35^, ${v}_{21}$ is the radiation frequency, and ${z}_{21}$ is the dipole matrix element of ULL 1 and LLL 2^36^. It is expected to set ${f}_{21}$ at a considerably large value to reach higher gain. The LLL 2 is depopulated by the extraction level (EL) 3, and the electrons are then relaxed into IL 4 quickly by LO-phonon scattering, and afterwards injected into the next period. The phonon well is always widest to adjust the separation between EL 3 and IL 4 (~ 92.4 meV) slightly larger than LO-phonon energy in GaN. The lifetime of ULL 1 is much longer than LLL 2 and thus guarantee population inversion.

**Figure 2:**
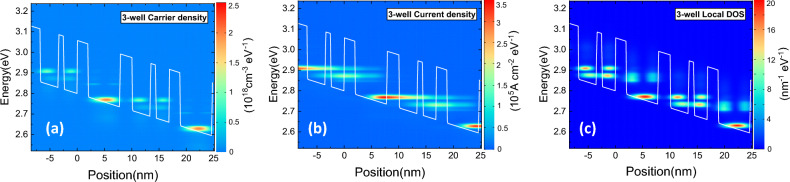
The 2D maps of position-energy resolved (**a**) carrier density, (**b**) current density, and (**c**) local density of states (DOS) as function of energy and position at 10 K under operating bias of 135 mV/period for the three-well RP structure.

Figure 2 depicts the 2D maps of position-energy resolved carrier density, current density, and local density of states (DOS) at 10 K under operating bias of 135 mV/period. IL 4″ and ULL 1 are aligned, and the population inversion between ULL 1 and LLL 2 has been established. The LLL 2 is depopulated through resonant tunneling, and the anticrossing energy between LLL 2 and EL 3 is 5.1 meV. The EL 3 is deliberately shifted down by 6–9 meV at around the operation bias to suppress the direct tunneling from IL 4″ and ULL 1 to EL 3, taking into account the severe broadening of this excited level due to the intrinsic strong e-phonon coupling in GaN. We notice the slight leakage through the extraction barrier, which can be further inhibited by thickening this barrier.

**Figure 3:**
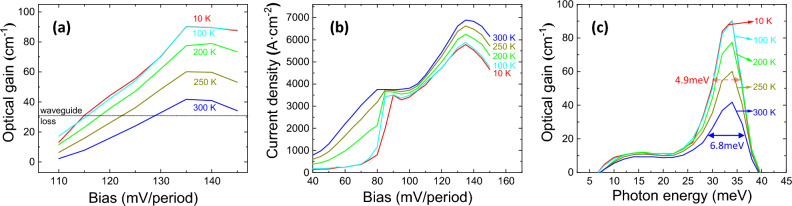
The calculated (**a**) maximum optical gain, (**b**) current density as functions of applied bias, and (**c**) gain spectra as functions of photon energy, in three-well designs from 10 to 300 K.

Figure 3 demonstrates the calculated (a) maximum optical gain, and (b) current density as functions of applied bias, and (c) gain spectra as functions of photon energy from 10 to 300 K. A peak gain of 90.1 cm^−1^ is obtained under bias of 135 mV/period at 10 K, and maintains 41.8 cm^−1^ at 300 K, which is above the estimated total cavity loss (20–30 cm^−1^), revealing the possibility of lasing at room temperature. The notorious non-radiative transition caused by thermally activated LO phonon emission for a GaAs THz-QCL plays a relatively small role for nitrides since the GaN LO-phonon energy is much larger^37^. The threshold current density is 4.3 kA cm^−2^ at 10 K and rise to 6.5 kA cm^−2^ at 300 K. Figure 3c shows that the photon energy at maximum gain is 34 meV, corresponding to 8.2 THz, and the linewidth of the gain spectra exhibited a slight increase from 4.9 meV at 10 K to 6.8 meV at 300 K.

### Two-well PSI scheme

In the three-well RP design, carriers are injected to ULL by resonant tunneling, thereby setting a limitation of maximum population inversion of 50% of the total amount of electrons. For THz-QCL, the separation between ULL and LLL is small, restricting the selective injection efficiency^38, 39^. In the recently reported two-well direct-phonon scheme, which achieved an impressive operation temperature of 250 K^5^, the LLL is the first excited state in phonon well and is directly depopulated by phonon scattering. However, unfortunately, it is not applicable for GaN based THz-QCLs to use any excited state as a lasing level, since such an excited state in the GaN phonon well is significantly broadened due to the much stronger e-phonon coupling strength^9^. Instead, two ground states can be used as lasing levels. In such a way, the first excited state plays a role for extracting and injecting carriers into the ULL by phonon scattering. This is the well-known PSI scheme^10, 15–18, 20, 21, 40, 41^. Therefore, the two-well PSI scheme is used here. Both ULL and LLL are the ground states, avoiding the broad excited states. In addition, it only requires one resonant tunneling between LLL and EL, which releases the strict tunneling alignments in the RP scheme. One must notice that such a two-well PSI scheme has been experimentally realized very recently by our group^17^.

**Figure 4:**
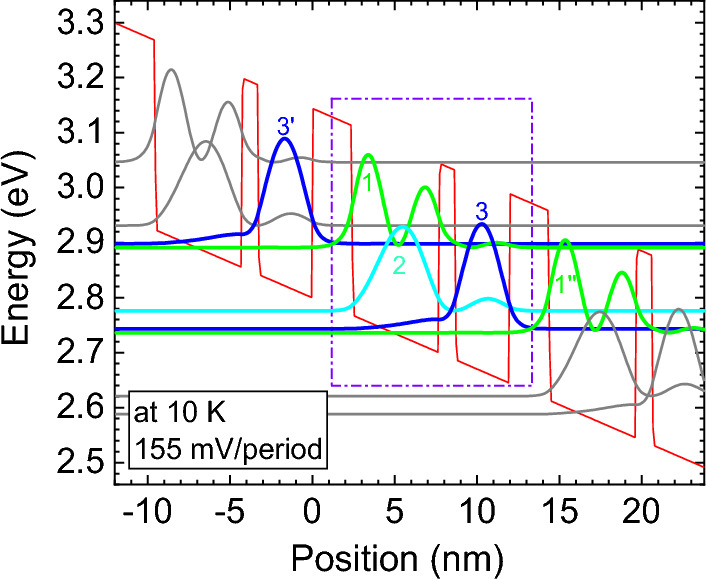
Conduction band diagram and envelope wavefunctions of TB subbands of the two-well PSI GaN THz-QCLs. The operating bias is 155 mV/period at 10 K. A tight-binding mode is applied at injection barrier between two adjacent periods. The thicknesses of the quantum well (GaN) and barrier (Al_0.2_Ga_0.8_N) for each period are 24.2/52.6/10/32.8 Å (barrier thicknesses in bold). The periodic sheet doping density is set to 6 × 10^10^ cm^−2^ with the 5-nm-wide doping in the middle of the widest well.

The two-well PSI design employs Al_0.2_Ga_0.8_N and GaN as the barrier and well. A higher Al composition of 20% is adopted to inhibit leakage from ULL to EL with stronger constraint^18, 32^. The layer sequence from the injection barrier is 24.2/52.6/10/32.8 Å with the barriers in bold fonts. The sheet doping density is 6 × 10^10^ cm^−2^ and the doping region is 5 nm wide in the middle of the widest well, and CBO is 343 meV. The conduction band diagram and the TB wavefunctions of the 3 lowest levels are shown in Fig. 4 at the operating bias of 155 mV/period. The operating voltage is ~ 20 mV/period higher than the three-well design due to the larger separation between the first excited state and ground state caused by the narrower phonon well. Such a separation is deliberate, about 20 meV larger than the GaN LO phonon energy, for the purpose of reducing the too strong phonon scattering in order to suppress the carrier leakage directly from the ULL to the EL, and then to the next ULL via the fast phonon scattering.

First, electrons are extracted by the EL 1 through extraction barrier. Next, electrons are relaxed into ULL 2 due to phonon scattering, and such LO-phonon scattering mechanism is quite swift. Thereafter, the transition between ULL 2 and LLL 3, with an oscillator strength of 0.34, emits a photon of corresponding frequency. Finally, the depopulation of LLL 3 is realized by EL 1″ of the next period, and this process is also fairly fast, resulting in a considerable amount of population inversion (~ 3.17 × 10^10^ cm^−2^). Compromise for the width of barriers must be made in this structure. A too thick extraction barrier will deteriorate the extraction efficiency from LLL 3 to EL 1″, and otherwise will lead to direct leakage from ULL 2 to EL 1″. Meanwhile, a too thick lasing barrier will reduce the wavefunction overlap between ULL 2 and LLL 3, and reversely, it may increase the leakage from ULL 2 and EL 1″ as well. Due to the proposed thicker and taller extraction barrier, it is recognized weaker coupling between LLL 3 and EL 1″ with a small anticrossing energy (3.1 meV). Simultaneously, LLL 3 preserves comparatively large distance from ULL 2 (~ 31.7 meV), which inhibits current leakage.

**Figure 5:**
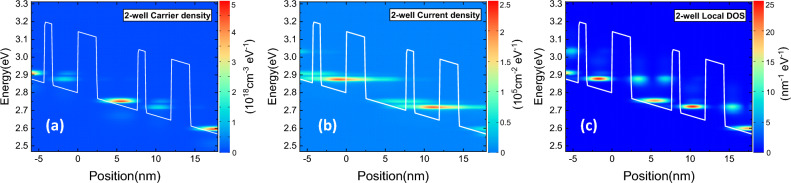
The 2D maps of position-energy resolved (**a**) carrier density, (**b**) energy differential current density, and (**c**) local DOS for the two-well PSI structure at 10 K under operating bias of 155 mV/period. Different from previously discussed in three-well design, the population inversion is much clearer and current leakage from ULL 2 to EL 1″ of neighboring period is obviously suppressed.

Figure 5 shows the 2D maps of position-energy resolved carrier density, current density and local DOS for the two-well PSI structure at 10 K under operating bias of 155 mV/period. Different from previously discussed in three-well design, majority carriers occupy the ULL 2, the population inversion is much clearer, and current leakage from ULL 2 to EL 1″ in the next period is obviously suppressed as seen in Fig. 5b.

**Figure 6:**
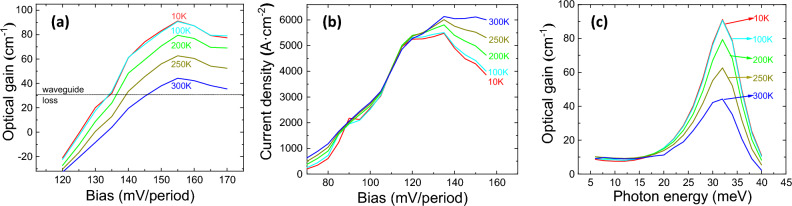
The calculated (**a**) maximum optical gain, (**b**) current density as a function of applied bias, and (**c**) gain spectrum as a function of photon energy, in two-well designs from 10 to 300 K.

Figure 6 demonstrates the calculated maximum optical gain, current density as functions of applied bias, and gain spectra as functions of photon energy for the two-well PSI design from 10 to 300 K. Peak gain of 91.3 cm^−1^ appears under operating bias of 155 mV/period at 10 K, and still 44.2 cm^−1^ for 300 K, which is promising for non-cooling system. The threshold current density is 5.4 kA cm^−2^ at 10 K and rise to 6.0 kA cm^−2^ at 300 K. Besides, the photon energy at maximum gain is 32 meV, corresponding to 7.7 THz, as seen in Fig. 6c.

### Doping effects for both schemes

**Figure 7:**
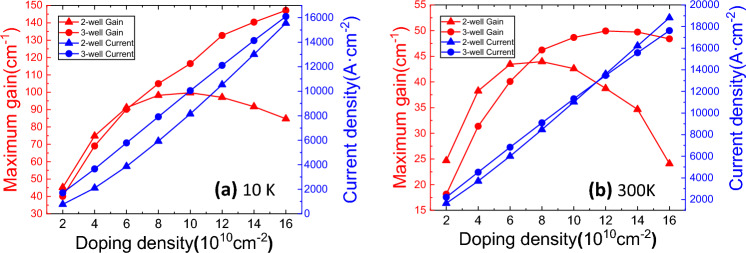
The maximum gain and current density as functions of doping density (**a**) at 10 K, and (**b**) at 300 K.

In order to find the suitable doping, simulations have been conducted in a wide doping density range for the two structures. The screened Coulomb potential based on the Debye model is used to calculate the self-energy of the charged impurity scattering ^19^. As seen in Fig. 7a, the peak gain at 10 K increases monotonically with doping in the three-well RP structure, reaching 146.2 cm^−1^ at 1.6 × 10^11^ cm^−2^. In contrast, the peak gain attains an upper limit of 99.7 cm^−1^ at doping density of 1.0 × 10^11^ cm^−2^ for the two-well PSI structure. The current densities of two structures both increase with doping density. Figure 7b demonstrates the peak gain and current density of the two structures at 300 K. For the two-well structure the peak gain saturates at around 42–44 cm^−1^ when doping density is 6–10 × 10^10^ cm^−2^. For the three-well structure, the peak gain also saturates, reaching a maximum value of 50.0 cm^−1^ at about 1.2 × 10^11^ cm^−2^. Both structures show maximum peak gain at a lower doping density at room temperature, but still preserve the possibility of lasing.

**Figure 8:**
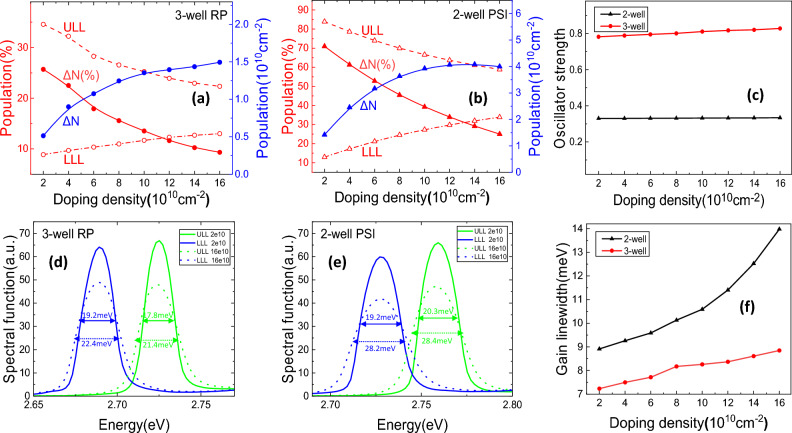
(**a**,**b**) The percentage of population and the absolute population inversion density of both structures. (**c**) The oscillator strength between ULL and LLL as a function of doping density for both structures. (**d**,**e**) The spectral functions of the ULL and LLL at doping density of 2.0 × 10^10^ and 1.6 × 10^11^ cm^−2^ for both structures. (**f**) The FWHM of the gain spectrum as a function of doping density for the two structures. All are at 10 K.

To understand such a distinct dependence of the peak gain on doping density, we have to check the important parameters. The peak gain centered at ${v}_{0}$ is $g({v}_{0})={\Delta N}_{21}{e}^{2}{f}_{21}/2\uppi {m}_{e}^{*}cn\Delta v{\varepsilon }_{0}$, where ${\Delta N}_{21}$ is the three-dimension population inversion, $c$ is the speed of light in vacuum, $n$ is the effective index of the mode of interest, $\Delta v$ is the linewidth of radiative transition, and ${\varepsilon }_{0}$ represents the dielectric permittivity of vacuum. The population inversion, oscillator strength, and broadening at 10 K are plotted in Fig. 8. In terms of population inversion, the percentage of population inversion (red solid lines in Fig. 8a,b) shows roughly same downward trend for both structures. This is due to the reduction of the ULL population and the increase of the LLL population with increasing doping density, as shown by the red dashed and dotted lines, respectively. Such a ΔN(%) reduction can be partly attributed to the stronger scattering by more impurities and electrons, and as well as the distortion of the band profile due to the doping induced band bending, which results in deviation from the resonant tunneling conditions and thus carrier redistribution^23, 26^. The density of population inversion of the two-well PSI structure basically maintains about 2.7–2.9 times as that of the three-well RP structure.

On the other hand, as shown in Fig. 8c, although the oscillator strength $f$ of the two-well PSI structure is much smaller than that of the three-well in high doping density, their ratio only slightly changes, 0.33/0.78–0.33/0.83. Therefore, the reason for the distinct gain decreasing as doping increases for the two structures can be inferred as the distinct broadening behavior as a function of doping.

The spectral functions of the ULL and LLL of the two structures are shown in Fig. 8d, )at doping density of 2.0 × 10^10^ and 1.6 × 10^11^ cm^−2^. The two-well PSI structure suffers a more severe broadening $\Delta v$, which is caused by the electron-impurity scattering and electron–electron scattering with increasing doping density. Consequently, as demonstrated in Fig. 8f, the FWHM of the gain spectra for the two-well structure increase more sharply, namely much wider gain spectra and resulting in severe drop of the peak gain as doping increases as shown in Fig. 7. Take 1.6 × 10^11^ cm^−2^ as an example, putting the three relevant quantities into the equation, $\frac{{g}_{3}}{{g}_{2 }}=\frac{\Delta {N}_{3}\cdot {f}_{3}/{\Delta v}_{3}}{\Delta {N}_{2}\cdot {f}_{2}/{\Delta v}_{2}}=\frac{1.51\times {10}^{10}\times 0.83/8.4}{3.99\times {10}^{10}\times 0.33/14.0} \approx 1.6$, which matches with the ratio of the peak gain values in Fig. 7a. Combined the detailed analysis of the three important parameters, the distinct peak gain behavior of the two structures can be well understood.

## Conclusion

We have theoretically investigated M-plane GaN based three-well RP and two-well PSI THz-QCL structures and calculated carrier transport, current density and gain properties through a self-consistent NEGF method. Both structures show peak gain of 90.1 and 91.3 cm^−1^ at 10 K, and 41.8 and 44.2 cm^−1^ at 300 K with a doping density of 6 × 10^10^ cm^−2^, all above the total cavity loss. The gain spectra indicate that lasing can happen at 8.2 and 7.7 THz, which are both beyond the conventional GaAs based THz-QCLs. Furthermore, the doping effect on the two designed structures has been investigated. The peak gain of the three-well structure increases monotonically with doping, but that of the two-well structure saturates at 1.0 × 10^11^ cm^−2^ at 10 K. The gain saturation of the two-well structure is mainly attributed to much more severe broadening of the lasing levels induced by electron–impurity and e–e scattering, and thus much broadened gain spectra. The results indicate that lasing of m-plane GaN THz-QCLs at unprecedented frequency at 300 K is possible.

## Data Availability

The datasets analyzed in the current study are available from the corresponding author on request.
